# Efficient ship noise classification with positive incentive noise and fused features using a simple convolutional network

**DOI:** 10.1038/s41598-023-45245-6

**Published:** 2023-10-20

**Authors:** Xu Lin, Ruichun Dong, Yuqing Zhao, Rui Wang

**Affiliations:** 1 College of Ocean Science and Engineering, Shandong University of Science and Technology, Qingdao, 266590 China; 2 College of Mechanical and Electronic Engineering, Shandong University of Science and Technology, Qingdao, 266590 China

**Keywords:** Physical oceanography, Acoustics

## Abstract

Ship noise analysis is a critical area of research in hydroacoustic remote sensing due to its practical implications in identifying ship direction, type, and even specific ship identities. However, the limited availability of data poses challenges in developing accurate ship noise classification models. Previous studies have mainly focused on small-sample learning approaches, resulting in complex network structures. Nonetheless, underwater robots often have limited computing power, making it essential to develop simpler recognition networks. In this paper, we address the issue of data scarcity by introducing positive incentive noise. We propose a CNN-based hydroacoustic signal recognition method that achieves comparable or superior performance to previous studies, using a simple network structure as a back-end decision system. We describe the feature extraction process using a dataset with added noise and compare the performance of various features. Additionally, we compare our proposed method with previous studies. Experimental results demonstrate that simple neural networks can achieve high performance and excellent generalizability without the need for complex network structures like adversarial learning models.

## Introduction

Hydroacoustic signals are the primary means of long-range communication in the ocean, and ship noise identification is essential in analyzing these signals. Ship-radiated noise, which refers to signals generated by ships and received by passive sonar systems, is widely used for ship target identification^1^. How to carry a ship identification system on an autonomous underwater robot has become one of the directions of interest for researchers. However, due to the complexity of the marine environment and the difficulty of data collection, identifying ships through hydroacoustic remote sensing is challenging.

To address these issues, various signal processing methods have been proposed for extracting features of hydroacoustic signals, including LOFAR spectra, Meier scalar spectrograms, Meier cepstral coefficients (MFCC), and Hilbert-Huang transform features^2^. With the development of deep learning, features based on these methods have been used to develop ship signal identification models^3–5^. However, the existing hydroacoustic datasets are limited, and different recognition methods mainly compete for minor sample problems, which must be more generalizable.

Some scholars input raw audio directly into the neural network for recognition. Hu et al.^6^ input raw audio into the CNN mesh layer to improve the network's generalization ability. Shen et al.^7^ developed an auditory-inspired convolutional neural network incorporating multi-scale expansion to enhance its generalization capabilities.

More scholars have used spectral features as input to the network, and the research direction has shifted to feature enhancement and small sample learning problems.

Mishachandar and Vairamuthu^8^ proposed a marine noise classification and recognition system using MFCC as input, capable of classifying unidentified marine sounds from cetaceans, fish, marine invertebrates, anthropogenic sounds, natural sounds, and passive acoustic marine noise recordings. Liu et al.^9^ connected the Meier spectrum with first- and second-order derivatives to increase the input bits of features and used recurrent neural networks for recognition. Ibrahim et al.^10^ introduced migration learning to grouper sound classification and demonstrated that migration learning has good recognition accuracy. Sun et al.^11^ introduced convolutional neural networks to multi-target recognition of hydroacoustic signals. They demonstrated that using amplitude Short Time Fourier transform^12^ (STFT), complex-valued STFT spectra, and log-mel spectra as network inputs could effectively recognize multi-target signals.

The computing chips on autonomous underwater robots are often unable to withstand the high arithmetic power, so developing simple high-performance networks has also become one of the difficulties.

To improve the effectiveness of recognition networks, data enhancement in image recognition can be employed. In this paper, a deep learning solution based on convolutional neural networks is proposed to improve data augmentation and network decision layer to improve network recognition accuracy using feature fusion. To explore the best hydroacoustic signal features, the original audio signal, MFCC, and different fused features are compared as inputs to the CNN network. To verify the generalizability of the network, the completed training network is applied to the new dataset DeepShip, achieving better results.

The contributions of this paper can be summarized as follows.

Firstly, unlike previous denoising methods, this paper proposes using positive incentive noise to introduce noise into the dataset extension and extract fused features as network inputs. Experimental results demonstrate that noise can improve the network recognition rate under certain conditions. This approach provides a novel solution to the issue of limited hydroacoustic datasets and improves the generalizability of ship noise classification models.

Secondly, the paper borrows the voting mechanism from random forest and combines it with CNN to add a decision layer at the back end of the CNN. This approach improves the accuracy of the network by fusing the output from multiple CNN models. This method contributes to the development of more robust and accurate ship noise classification models.

Lastly, the paper compares the existing algorithms with the proposed method and verifies that a simple network can also achieve high recognition accuracy. This finding is significant because it suggests that complex network structures, such as those involved in adversarial learning, may not always be necessary for accurate ship noise classification.

Section "Dataset setting" discusses the dataset setup and compares the enhanced dataset. Section "Methods" describes the design and parameters of the network. Section "Experiments" performs an experimental comparison to show the method's superiority.

## Dataset setting

In this paper, A dataset is produced based on the ShipsEar dataset^13^. This dataset collects the sounds of different vessels along the Spanish Atlantic coast during 2012 and 2013.

### Labels

To facilitate the study, 11 types of ship noise and one type of environmental noise are classified into five categories based on the classification of the ShipsEar dataset, as shown in Table 1. The network is still classified according to 12 categories.

**Table 1:** Labels.

Class ID	Name	Frames
A	Fishboat	142
	Tugboat	54
	Trawler	54
	Mussel boat	128
	Dredger	82
B	Motorboat	215
	Sailboat	42
	Pilot ship	121
C	Passengers	873
D	Ocean liner	187
	RORO	275
E	Natural ambient noise	319

Since the length of the noisy audio varies for each type of vessel in the dataset, the audio was uniformly sliced at every 200 ms to form the original dataset.

### Dataset expansion

When counting the data in the five categories, the audio dataset contained 375 audios in Category A, 310 audios in Category B, 852 audios in Category C, 321 audios in Category D, and 229 audios in Category E. To avoid discarding any audio, the slices with blank audio less than 80 ms were kept. To increase the data volume, we aim to expand the dataset efficiently and improve the network. We adopt Li’s latest proposal of positive incentive noise^14^, which is defined as follows.

Define the information of task $T$ with noise $f$:

1 $$MI\left(T,f\right)=H\left(T\right)-H(T|f)$$

where $MI$ is the mutual information and $H$ is the information entropy.

Define the noise $f$ that satisfies the following conditions as the positive incentive noise:

2 $$MI\left(T,f\right)>0$$

The above inequality is also equivalent to

3 $$H\left(T\right)>H(T|f)$$

On the contrary, the noise that satisfies $MI\left(T,f\right)=0$ is called pure noise or negative noise.

In contrast to the idea that noise always affects network accuracy, he argued that noise is not always harmful and that the effect of positive incentive noise on simple neural networks is more substantial than adversarial learning in some areas. Li concludes from Stochastic Resonance analysis^15^ that random noise is positively incentive in some data and pure noise in other cases, which means that there cannot be only pure noise on the data and not only pure positive incentive noise. This inspiration has led us to a unique perspective. While denoising is conventionally employed to handle datasets plagued by substantial ambient noise, introducing white noise could enhance the network's recognition rate. It's crucial to highlight that this approach diverges from the traditional practice of adding noise to data primarily for dataset enrichment. Instead, our process involves actively incorporating white noise to boost network performance, constituting a somewhat distinct conceptual twist on the idea.

The process of ship noise classification in machine learning typically involves the conversion of raw audio signals into spectrograms to extract relevant features for recognition. However, this conversion process may result in the loss of information from the original signal. Additionally, the filter used for creating the spectrogram may selectively retain certain signal features based on their frequency. To address these issues, a novel approach is proposed in this paper, which involves introducing random noise to the original audio signal prior to spectrogram conversion. The hypothesis is that the added noise will enhance the retained features after filtering, without affecting the feature extraction process, as the filter will effectively remove any extraneous noise. This hypothesis will be verified through a simple experiment.

To expand the dataset efficiently and improve feature extraction, random noise is added to all 2087 original slices, and a new dataset is formed with all audio without added noise, resulting in a dataset twice the size of the original data.

It is important to note that the number of class C data is significantly larger than the other four classes, and the amount of ambient noise data belong to class E is limited. To address this issue, a new dataset called DeepShip is introduced. DeepShip is open source on GitHub (https://github.com/irfankamboh/DeepShip) and only comprises four categories of ships(Cargo, Passenger, Tanker, Tugboat). The DeepShip part of the audio is sliced for the same plus noise processing according to the classification of ShipsEar to obtain a more balanced dataset. Finally, the ambient noise data is expanded through simulation to obtain the final dataset.

### Comparison experiment

The positive incentive noise experiment proposed by Li focuses on image recognition, so we add random noise to the original audio for experimental comparison.

The experimental parameters were set as follows in this study. The raw audio was uniformly converted into a STFT-spectrum extracted feature input to a CNN network for recognition, which consisted of two convolutional layers, two maximum pooling layers, an additional average pooling layer, and a fully connected layer in front of the output layer. The activation function was set to the common Relu function^16^, the optimizer to Adam^17^, and the network was trained for 100 epochs.

**Figure 1:**
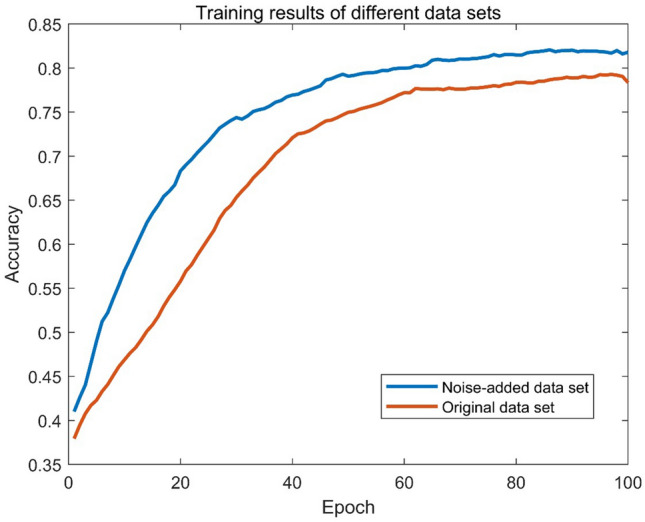
Recognition rate of original and noise-added data sets.

The most straightforward CNN network was used in this experiment to verify the noise improvement on the training results. The experimental results are shown in Fig. 1.

The experiment shows that the recognition accuracy of the original unnoticed dataset is only 78%, while the recognition accuracy of the dataset with random noise added reaches 80.2%. This proves that positive incentive noise is also effective for audio recognition.

## Methods

The traditional way of underwater target identification is through the human ear, and the method requires an operator with extensive experience and skilled hydrophone operation skills. When the operator captures the suspicious sound, adjusts the volume and filter, removes the background noise, and then analyzes the timbre, rhythm, and other acoustic characteristics to identify the target based on experience. Although the manual identification method has high accuracy, the required time and economic cost are more expensive.

The machine learning neural network represented by CNN enables the dimensionality of features to learn different acoustic target features by deepening the number of layers.

For the original audio, spectral filtering can extract most acoustic features. For example, the Meier spectrum is similar to the human ear for low-frequency sensitivity and can retain most acoustic features. However, different spectrums extracting features must lose some features simultaneously, so we propose using different feature extraction methods for feature fusion and then input to the network for recognition.

**Figure 2:**
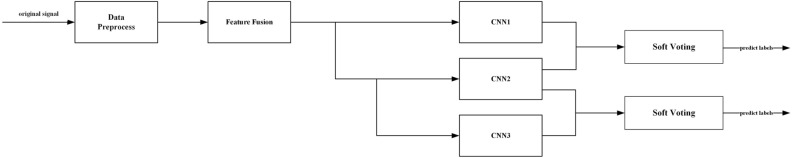
Overall framework.

The framework is as follows, firstly, different features are extracted from the original audio, and then the features are fused and fed into a deep convolutional network. The back-end decision can effectively improve the recognition accuracy, so we use a convolutional network for further feature extraction of the fused features. Then, feeding multiple neural networks for parallel training and adding a voting decision mechanism at the back end, the detailed process is shown in Fig. 2.

### Feature extraction

The Mel cepstral coefficient MFCC is a feature widely used in speech signal recognition and was introduced by Davis and Mermelstein^18^ in the 1980s. The Mel scale describes the nonlinear properties of human ear frequencies, which are related to linear frequencies as in Eq. (4):

4 $$Mel\left(f\right)=2595\mathrm{lg}(1+f/700)$$

where $f$ is frequency.

The specific steps of MFCC feature extraction on this basis are shown below:

Step (a): Pre-emphasis, boosting the high-frequency part to stabilize the features. Since the high-frequency part of the signal attenuates much more than the low frequency during underwater propagation, the high-frequency part of the signal will be ignored if feature extraction is performed directly. Therefore, pre-emphasis can effectively extract the stable signal features.

5 $$H\left(z\right)=1-\mu {z}^{-1}$$

where H is the pre-emphasized signal, z is the original signal, and μ is the pre-emphasis coefficient, generally considered 0.9 ~ 1.

Step (b): Take the number of data points N = 2048 for each frame, and sub-frame the sampled noise sequence. In order to avoid too much variation between two adjacent frames, there is generally an overlap between two adjacent frames containing M sampling points, and M is taken as 512 in this paper.

Step (c): Adding windows, multiplying the Hamming windows in each frame to increase the continuity between the left and right sides of the frame. After multiplying the Hamming window $W(n)$, the speech signal ${S}{\prime}\left(n\right)$ can be expressed as

6 $${S}{^\prime}\left(n\right)=S(n)\times W(n)$$

7 $$W\left(n\right)=\left\{\begin{array}{c}0.54-0.45\times \mathrm{cos}\left[\frac{2\pi n}{N-1}\right],0\le n\le N-1\\ 0,\,\,\,\,\,\,\,\,\, other\end{array}\right.$$

Step (d): Fourier transform^19^, the Fourier transform of the signal after framing, and then the signal's spectrum is modulo and squared to obtain the signal's power spectrum. The fast Fourier transform of the signal is

8 $$x\left(k\right)=\sum_{n=0}^{N-1}x(n){e}^{\frac{-2j\pi k}{N}}$$

$x\left(k\right)$ is the amplitude spectrum, $x(n)$ is the input signal, and *N* denotes the number of Fourier transform points.

Step (e): Mel filter bank filtering, transferring the frequency domain f to the Mel domain for signal processing.

9 $${H}_{m}(k)\left\{\begin{array}{c}\frac{k-f\left(m-1\right)}{f\left(m\right)-f\left(m-1\right)},f(m-1)\le k<f(m)\\ 1, \,\,\,\,\,\,\,\,\,\,k=f(m)\\ \frac{f\left(m+1\right)-k}{f\left(m+1\right)-f\left(m\right)},f(m)<k\le f(m+1)\\ 0, \,\,\,\,\,\,\,\,\,\,other\end{array}, 0\le m\le L\right.$$

where $\sum_{m=0}^{M-1}{H}_{m}\left(k\right)=1$, ${H}_{m}(k)$ denotes the filter parameters, *f (m)* denotes the center frequency of the triangular filter, and *L* is the number of Meier filters.

The bandpass filter output *D(m)* is

10 $$D\left(m\right)=\sum_{k=0}^{N-1}{\left|X\left(k\right)\right|}^{2}{H}_{m}(k)$$

where N is the total number of signal points in each frame.

Step (f): The MFCC parameter C(n) is obtained after taking the logarithm of *D(m)* and then performing the discrete cosine transform

11 $$C\left(n\right)=\sum_{k=1}^{L}\mathrm{lg}\left(D\left(m\right)\right)\mathrm{cos}\left[\frac{\pi \left(k-0.5\right)n}{L}\right],n=\mathrm{1,2},3,\cdots ,p$$

where *p* is the MFCC order.

Another commonly used feature extraction method is the STFT spectrum^20^. The basic idea of STFT is to add a window to the signal and then perform the Fourier transform. The window function is translated throughout the time axis according to the change of time. That is, the spectrum near the moment t is localized using the window function, thus constituting a two-dimensional time–frequency spectrum of the signal to be analyzed.

12 $$STFT=\left(\omega ,\tau \right)={\int }_{-\infty }^{+\infty }g(t-\tau )s(t){e}^{-i\omega t}dt$$

where the time duration of the window function is t, and the center frequency of the window function is ω.

**Figure 3:**
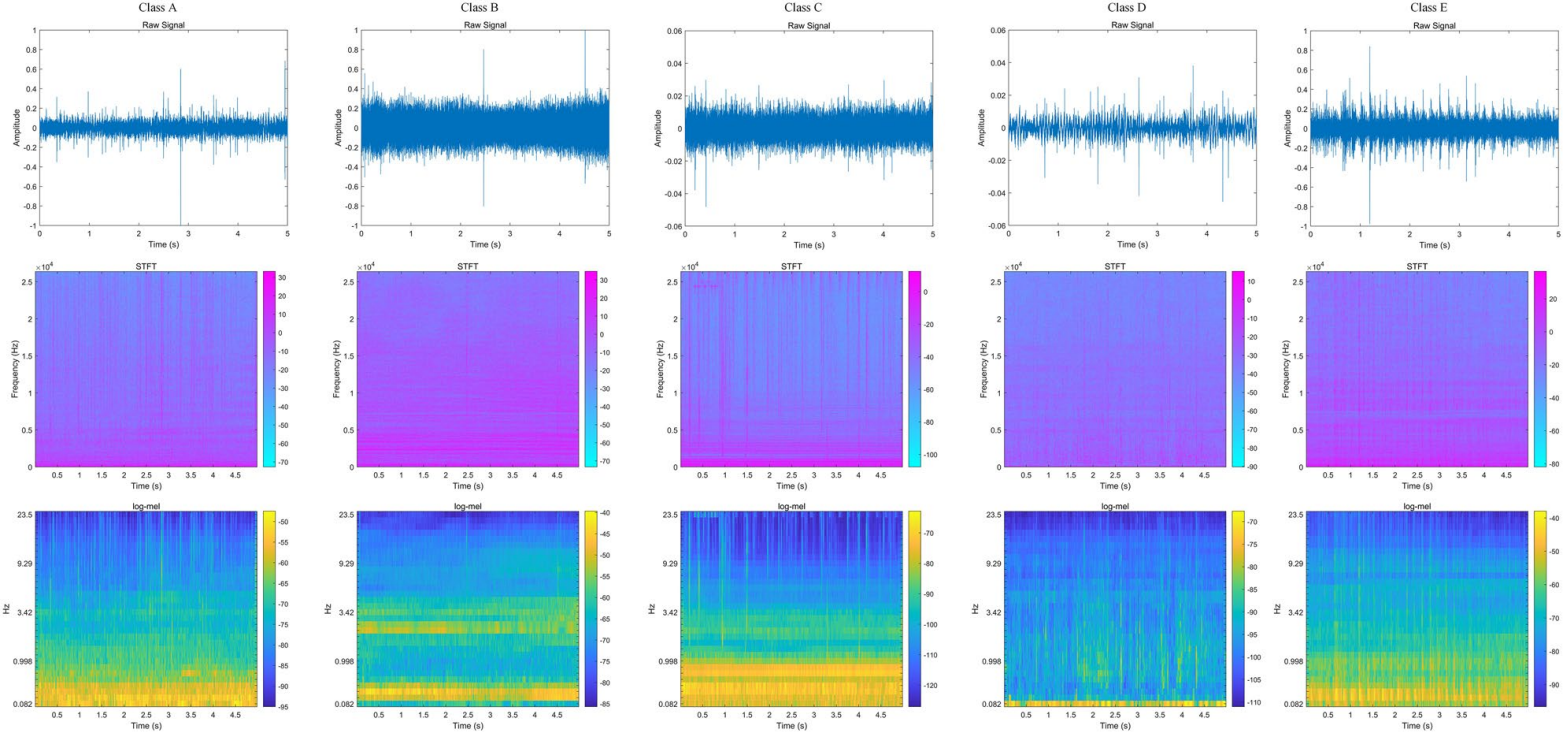
Examples of features. Row 1: original audio wav; row 2: magnitude STFT spectrogram; row 3: log-mel spectrogram.

From the principles of various types of feature extraction methods, it can be seen that each method occurs with partial loss of signal features. We want to enhance the extracted features while preserving their features as much as possible. Figure 3 shows the spectra of various feature extraction methods for five types of noise.

In this paper, The librosa software package^21^ is used for data processing and feature extraction. Referring to the idea of the Mel spectrum connected with first and second-order derivatives used by Liu et al.^9^, Extracting the STFT spectrum and logging the Mel spectrum connected to form a four-dimensional feature input to the CNN network for convolution.

### CNN parameters

In this study, drawing inspiration from the widely acclaimed CNN architecture ResNet^22^, we aimed to construct a streamlined CNN network that could attain exceptional recognition accuracy. To accomplish this, we crafted a straightforward CNN network, drawing influence from an alternative network structure known as DenseNet^23^. CNN networks can automatically extract features from raw data and learn high-level abstract features of the data for tasks such as classification and recognition through operations such as convolution and pooling.

Diverging from the complexity of other networks, such as Convolutional Recurrent Neural Network(CRNN), a CNN network with only two convolutions and a one-dimensional stratification pool before the output layer was used in this study. The detailed structure is shown in Tables 2 and 3. Figure 2 illustrates three CNN structures, where the parameters of network 3 differ from those of network 1 and network 2. Network 1 is identical to network 2, and network 3 was applied for comparison in experiment III.

**Table 2:** CNN1.

Layer	Size	Number of channels	Activation function
Conv2d	3 × 3	64	Tanh
Max_Pooling2d	2 × 2		
Conv2d	3 × 3	128	Tanh
Max_Pooling2d	2 × 2		
Dropout (0.1)			
Flatten			
Dense	1024		Tanh
Dense	12		Softmax

**Table 3:** CNN3.

Layer	Size	Number of channels	Activation function
Conv2d	3 × 3	32	Relu
Max_Pooling2d	2 × 2		
Conv2d	3 × 3	64	Relu
Max_Pooling2d	2 × 2		
Dropout (0.25)			
Flatten			
Dense	128		Relu
Dropout (0.5)			
Dense	12		Softmax

The original data were divided into different subsets, and each subset was used to train a CNN model. K-Fold cross-validation^24^ was used to divide the data into K copies, using K-1 copies each time as training data and the remaining copy as test data. This approach effectively reduces overfitting and provides better generalization ability. In this study, two CNN networks with the same parameters (shown in Fig. 3) were combined, and K was set to 2.

The fused features extracted in the previous section were used as the CNN network input, and the hyperbolic tangent function tanh was chosen as the activation function. After two layers of convolution and pooling, the classification probabilities of the 12 categories were output through the fully connected and softmax layers^25^.

In contrast to the VGG16 network^26^, often used for image classification tasks and includes 13 convolutional layers and three fully connected layers, the VGG network was initially proposed to solve large data sets. A simple network structure may be more practical in a small sample problem like ship noise with a few parameters and fast model convergence.

In Experiment I, different features were extracted for fusion and input into CNN1 for experiments. The aim was to verify whether the features retained by feature fusion could effectively improve the recognition accuracy of the network. No changes were made to the network structure.

In Experiment II, the recognition accuracy of the complex network was compared with that of the simple network proposed in this paper. The aim was to verify the conjecture that the simple network could achieve comparable or even better performance than the complex network.

### Voting mechanisms

In addition to using CNNs, we also considered the feasibility of a random forest decision-making approach for hydroacoustic remote sensing. Random forest^27^ is an integrated learning algorithm that improves prediction accuracy by integrating multiple decision trees (Decision Tree). Unlike CNNs, random forests are suitable for processing structured data and require manual feature selection and extraction.

There are familiar combination strategies for integrated learning, such as averaging and voting. Random forest is a typical integrated learning algorithm that uses a combination strategy of the voting method. It relies on the voting choice of each decision tree in the random forest to determine the final classification result. At the outset of our research, our primary focus was on studying the Random Forest algorithm. However, we consistently observed unsatisfactory recognition accuracy when applying Random Forest to hydroacoustic signal recognition, as demonstrated in Experiment II (refer to the experimental results). As we delved deeper into the Random Forest algorithm, we uncovered that its final decision layer typically employs a voting mechanism^28^. Moreover, we noted the substantial impact of the weighted soft-voting mechanism on enhancing the performance of deep learning models. Consequently, we integrated the weighted soft voting mechanism with our designed network to enhance the algorithm's performance.

**Figure 4:**
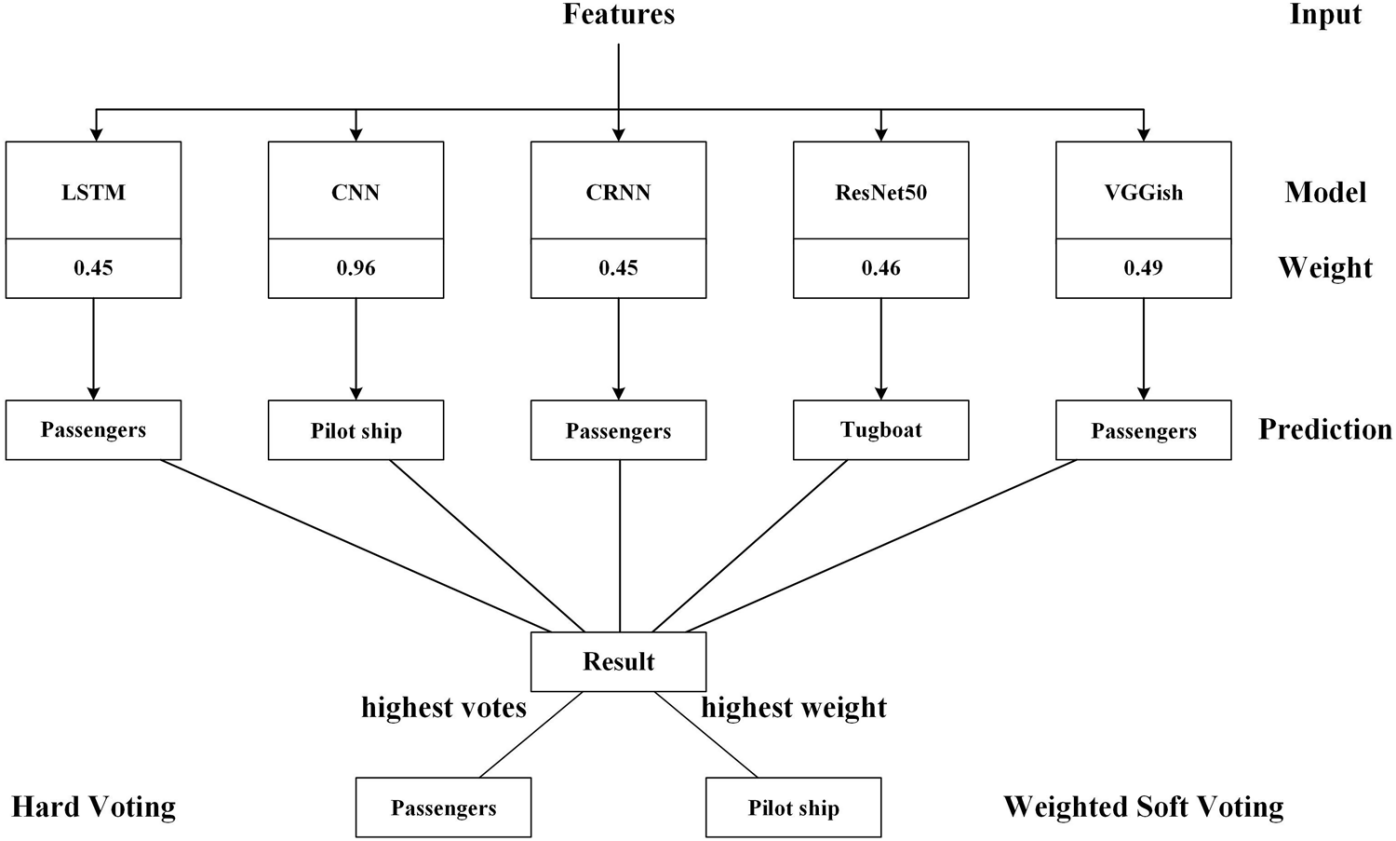
Soft voting. The picture shows the difference between soft and hard voting, not the prediction of the highest number of votes is the final result.

The standard voting methods are hard voting and soft voting. Hard voting is a simple mechanism in which multiple models' predictions are voted on, and the category with the most votes is ultimately selected as the prediction result. Soft voting^29^ is a probability-based voting mechanism known as weighted average probability voting. The prediction results of multiple models are considered probability distributions, and the final prediction result is a weighted average of the prediction probabilities of each model. Figure 4 shows the flow of the soft voting mechanism, and the figure visualizes the difference between soft voting and hard voting.

Due to the small sample problem of ship noise classification and using a simple network with the same parameters, weighted soft voting was chosen as the back-end decision mechanism in this study.

Experiment II compares the random forest with other neural network training results. In Experiment III, comparing the past studies with the present method. In Experiment IV, verifying the generalization ability of the method in this paper.

## Experiments

### Evaluation metrics

Ship noise classification is a multi-class classification problem. Since the number of data varies greatly between the various types of data in the dataset, using macro-averaged F1 scores to evaluate the classifier's performance.

The F1 score^30^ is calculated as follows.

13 $$F1=2\frac{(Precision*Recall)}{(Precision+Recall)}$$

*Precision* represents the proportion of true positive samples among the samples predicted as positive, and *Recall* represents the proportion of true positive samples correctly predicted as positive. The calculation formulas are as follows:

14 $$Precision=\frac{TP}{TP+FP}$$

15 $$Recall=\frac{TP}{TP+FN}$$

The macro-averaged F1 scores^31^ were averaged after calculating the F1 scores for each category without considering the difference in the number of samples in each category, i.e., the weights of each category were the same.

16 $${F1}_{macro}=\frac{1}{C}{\sum }_{i=1}^{C}{F1}_{i}$$

Also, the average precision (AP)^32^ has been selected, the area between the Precision-Recall curve and the coordinates, to evaluate the algorithm's performance.

17 $$AP={\int }_{0}^{1}precision(recall)d(recall)$$

### Experiment I

**Figure 5:**
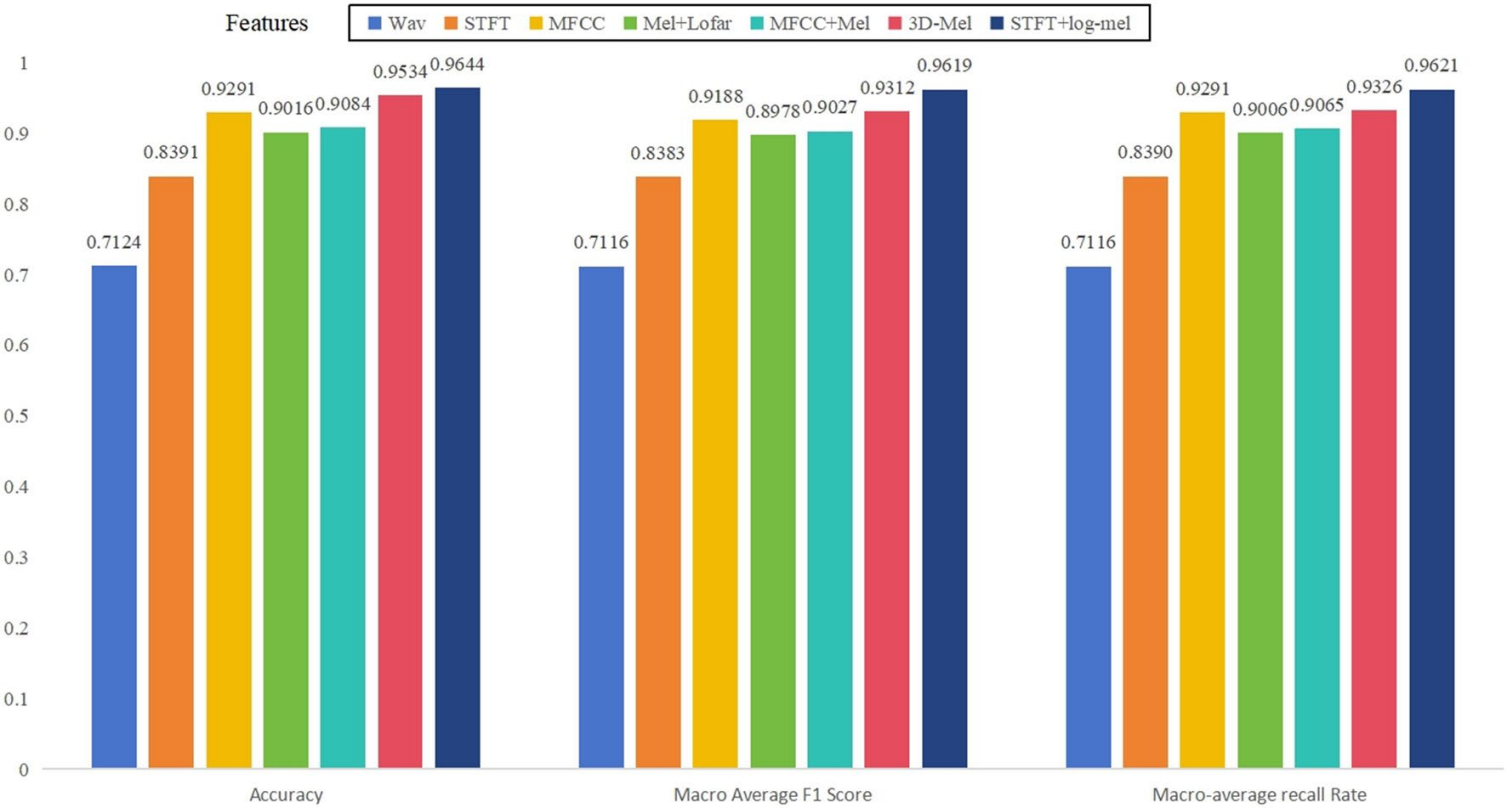
Partial results for different input features.

In Experiment I, we will use 75% of the data set for training and 25% for testing. It's worth noting that each class of ship data will be partitioned into a 75% training set and a 25% testing set. This approach aids in mitigating the imbalance in training outcomes resulting from variations in data volume among different classes, in contrast to a random data split. The different features are fed into the same network CNN1 for comparison. Figure 5 shows some identified features' accuracy, recall, and macro-average F1 scores.

In the original audio signal experiments, only 71.24% recognition accuracy was achieved in the test set. The accuracy only reaches up to 90% when using only a single spectrum as feature input. When trying to fuse multiple feature extraction methods, we find that the fusion of the Mel spectrum with first and second-order derivatives achieves 95% recognition accuracy, while using STFT with log-mel fusion slightly outperforms 3D-Mel^9^ with 95.34% recognition accuracy. Table 4 shows the results of all experiments.

**Table 4:** Recognition results of different features.

Feature	Accuracy (%)	Macro average F1 score	Recall rate (%)
Wav	71.24	0.7116	71.16
STFT	83.91	0.8383	83.90
MFCC	92.91	0.9188	92.91
Mel + Lofar	90.16	0.8978	90.06
MFCC + log-mel	90.84	0.9027	90.65
3D-Mel	95.34	0.9312	93.26
STFT + log-mel	96.44	0.9619	96.21

### Experiment II

In Experiment II, the idea of using a random forest to classify and recognize ship noise was validated. The same features(STFT + log-mel) were fed into the random forest for training and comparison with the CNN network.

Two models were used for the random forest parameter setting. The first was a random forest^33^ with no parameter changes, which contained 100 decision trees. The second was a random forest with Bayesian optimization^34^, which automatically found the optimal hyperparameters for the model. In both approaches, multiple models were trained in parallel for hard voting decisions. The experimental results are shown in Table 5.

**Table 5:** Comparison of random forest and CNN network training results.

Model	Accuracy (%)
Random forest	80.56
Bayesian optimized random forest	82.64
Integrated random forest	81.42
Integrated Bayesian optimized random forest	83.71
Soft voting CNN	97.34

From the experimental results, it is found that the random forest model performs poorly on the ship noise classification problem, but adding a voting mechanism can also improve the recognition accuracy of the model to a certain extent, which provides us with a new idea of adding a voting decision mechanism to the CNN network.

Finding the optimal hyperparameters of the random forest requires higher arithmetic power. In experiments, we found that the random forest model takes much longer to train than the simple network model in this paper.

### Experiment III

**Figure 6:**
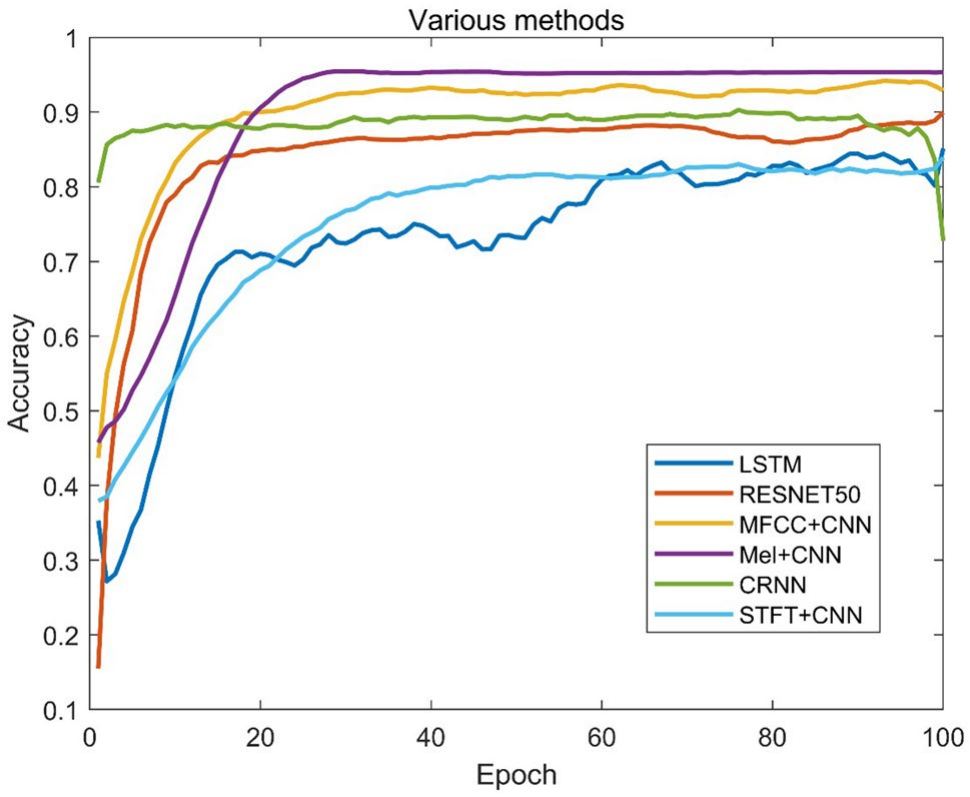
Accuracy curve of each method.

In Experiment III, the proposed method in this paper was compared with previous studies that used various types of features and adversarial learning networks. By replicating a previous study, we achieved 92.91% accuracy when using MFCC as the input to the CNN network and 95.64% accuracy when using 3D-mel as the feature. However, the network performance decreased when the STFT spectrum was used as the feature input. In this pa-per, migration learning was also performed using ResNet. The recognition accuracy curves for each method are shown in Fig. 6.

Table 6 shows the network structures and experimental results for each type of method.

**Table 6:** Training results for each method.

Model	Feature	Activation function	Macro f1 score	Recall (%)	Accuracy (%)
CRNN	MFCC	Relu	0.8842	88.46	90.27
LSTM	3D-Mel	Relu	0.8420	85.37	85.14
ResNet50	3D-Mel	Relu	0.8180	81.48	89.95
CNN1	MFCC	Tanh	0.9188	92.91	92.91
CNN1	STFT	Relu	0.8383	83.90	83.91
CNN1	3D-Mel	Relu	0.9312	93.16	95.34
CNN3	MFCC	Relu	0.9015	90.46	91.97
GAN	3D-Mel	Relu	0.9646	96.85	96.84
CNN + Voting	STFT + log-mel	Relu and Tanh	0.9719	96.19	98.44

The convolutional neural network (CNN) outperformed the convolutional recurrent neural network (CRNN) when using Mel-frequency cepstral coefficients (MFCC) as input, with a recognition accuracy of 92.91% under various CNN network parameters. In addition, CNNs with rectified linear unit (ReLU) activation function performed slightly worse than CNNs with a hyperbolic tangent (Tanh) activation function. However, when 3D-Mel was used as the network input, the recognition accuracy was significantly improved by 2.43%. A simple network consisting of fine-tuned ResNet50 weights trained on the ImageNet dataset did not produce satisfactory recognition results. In contrast, the adversarial learning network GAN achieved an impressive recognition accuracy of 96.84%. Interestingly, the composite CNN network with voting decisions did not differ much from the GAN in terms of recall and F1 score, but it achieved a higher recognition accuracy of 98.44%.

**Figure 7:**
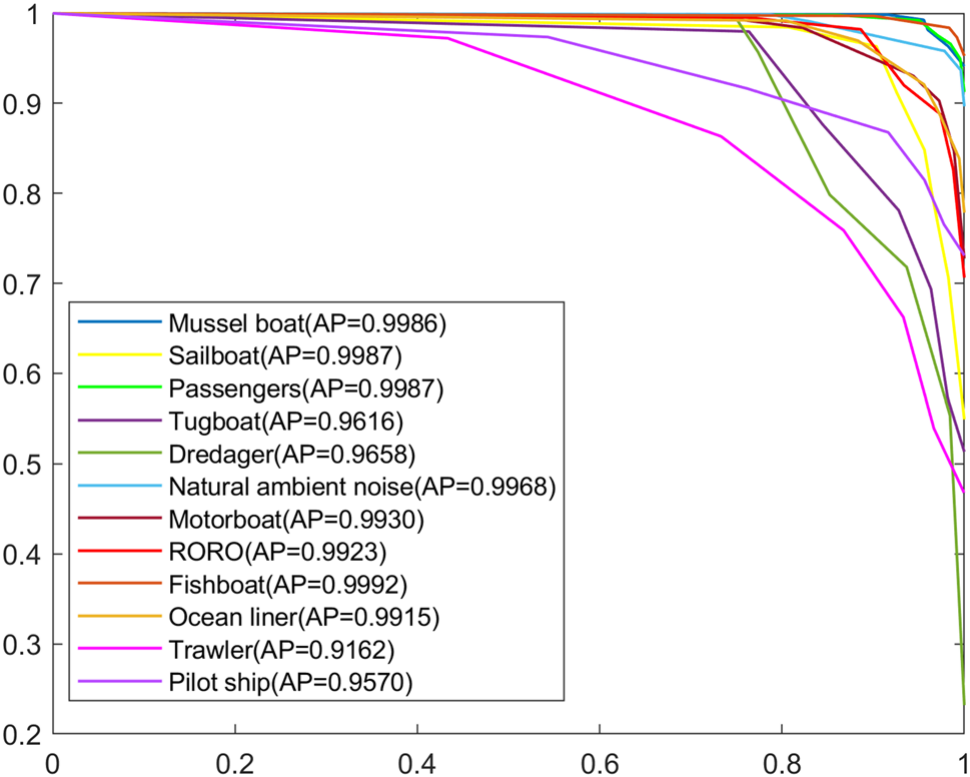
Precision–recall curves of each class.

**Figure 8:**
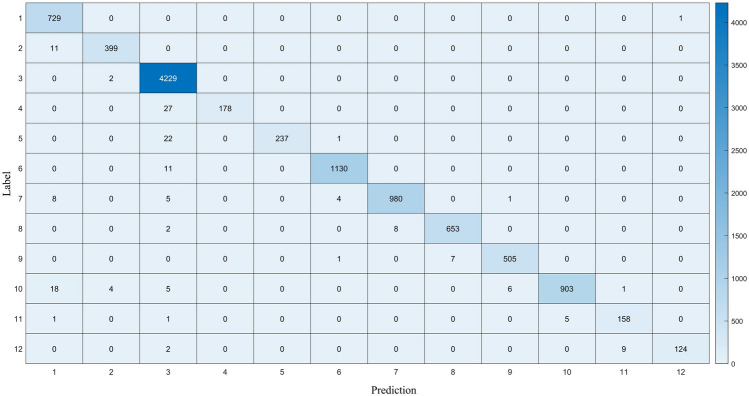
Confusion matrix. 1: Mussel boat; 2: sailboat; 3: passengers; 4: tugboat; 5: DredgER; 6: natural ambient noise; 7: motorboat; 8: RORO; 9: fishboat; 10: ocean liner; 11: trawler; 12: pilot ship.

Compared with previous studies, the method in this paper is more straightforward and has high performance. Figure 7 shows the Precision–recall (PR) curves of the method in this paper, and Fig. 8 shows the confusion matrix^35^ better to demonstrate the recognition results of 12 types of noise.

The PR curves for different types of noise show that the proposed method in this paper tends to achieve an average precision (AP) value close to 1.00, indicating high precision and recall values. However, due to the imbalanced data volume for certain types of noise, a few noise categories have relatively lower AP values. Overall, the PR curves demonstrate the effectiveness of the proposed method in accurately classifying ship noise, particularly in the presence of various types of noise.

Figure 8 shows that the noise with more data tends to have better training results, which lead to false recognition of the noise with fewer data.

### Experiment IV

**Figure 9:**
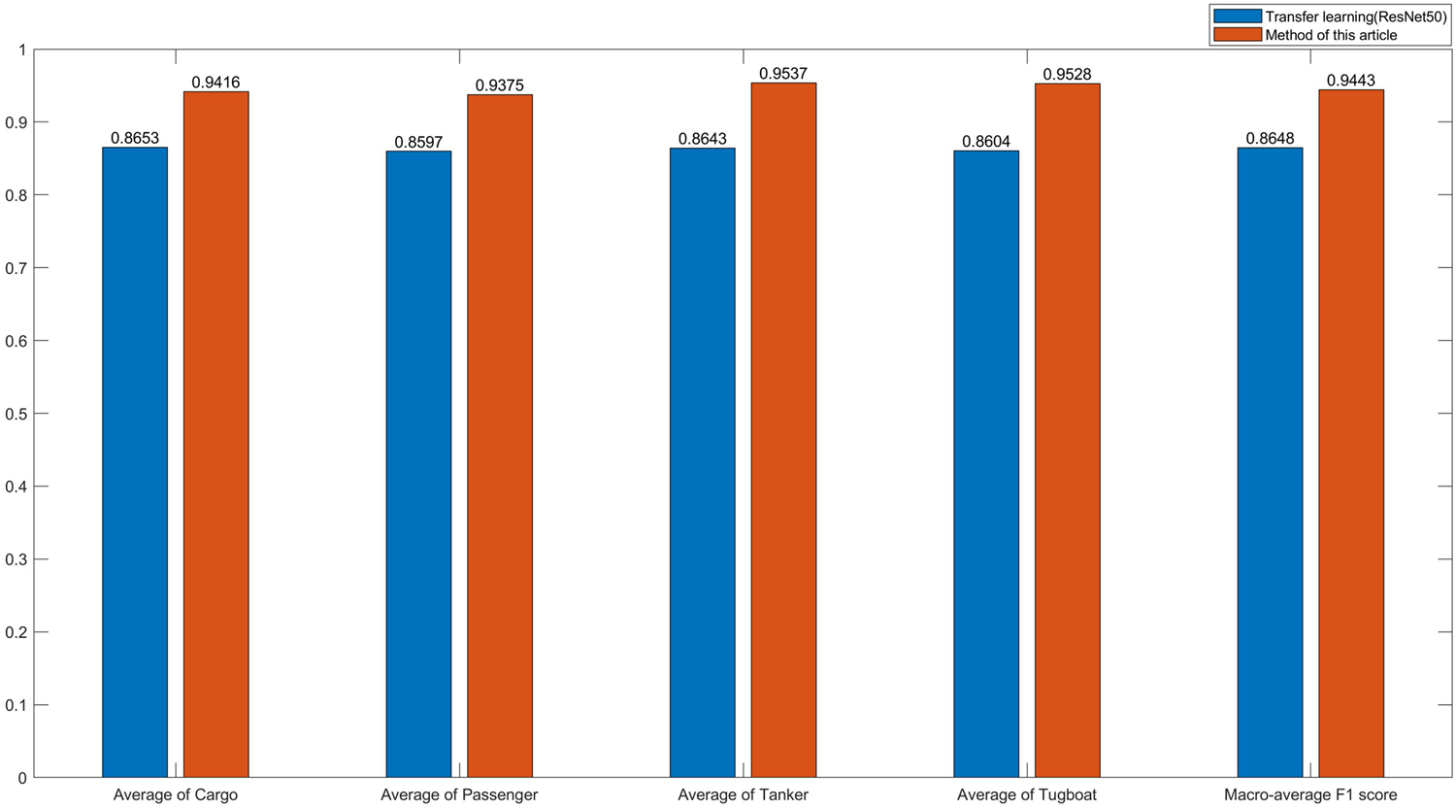
Identification results for the DeepShip dataset.

It is noteworthy that in Experiment I and Experiment II, the training and validation sets were separated due to the limited data volume. To evaluate the generalizability of our approach, we performed additional experiments with all DeepShip datasets as validation sets. The results were compared with those obtained using the Resnet50 network with migration learning, and Fig. 9 shows the experimental findings. Notably, the DeepShip dataset includes ships of the Tanker class, which is not present in the ShipsEar dataset. Therefore, we categorized Tanker as class D noise for our analysis.

The proposed method in this paper still has 94.47% recognition accuracy on the DeepShip dataset, and the comparison with the migration learning in Experiment III proves that the method has good generalization.

Since the DeepShip dataset contains only four types of ship noise, only two types of ship noise, A and C, as described in this paper, are included so that it impacts the test results.

## Discussion

The experimental results demonstrate that the choice of features used as network inputs in ship noise classification plays a decisive role in recognition accuracy, and that complex network structures may not always lead to im-proved accuracy. As various feature extraction methods can cause information loss, fused features tend to exhibit exemplary performance. The confusion matrix in Fig. 8 shows that data size still has an impact on the recognition results of the network.

In this study, weighted soft voting was used as the back-end decision-making method. Generally, the voting method should be used for different models to maximize the performance of each model. However, in cases where complex net-works are not always applicable, using the same network for decision-making may be more appropriate.

Although the training set used in this study was not separated from the test set, unlike previous studies, the method's generalizability was explored by applying the completed training models to the DeepShip dataset. It's important to highlight that the dataset utilized in this study was constructed as an extension of the original dataset. A noteworthy addition was the introduction of white noise as positive incentive noise to enhance the network's performance. Furthermore, it's crucial to acknowledge that the dataset lacked ambient noise data, and the ambient noise levels can significantly vary across different sea areas with distinct sea conditions. Consequently, the outcomes of ambient noise identification may not hold strong practical significance. Future research endeavors should focus on expanding the dataset to encompass diverse data sources. Additionally, we plan to incorporate more real data obtained from our own measurements in upcoming studies.

## Conclusions

This paper presents a novel approach to ship noise remote sensing classification using a simple CNN network structure. The paper introduces the concept of positive incentive noise, where the addition of noise can improve the recognition accuracy of the network. Additionally, the use of fused features as the network input leads to better performance than single features alone. The proposed method also utilizes a voting method in integrated learning to improve network performance without increasing complexity. Experimental results show that the network achieves a recognition accuracy of 98.44% and demonstrates better generalization ability compared to previous studies. Furthermore, the proposed method is validated on a new dataset, highlighting the effectiveness of using simple networks. Future research can focus on developing a method to accurately identify specific vessels.

## Data Availability

The original dataset used in this paper can be found at http://atlanttic.uvigo.es/underwaternoise/ and https://github.com/irfankamboh/DeepShip.
